# Alteration of prognostic efficacy of albumin‐bilirubin grade and Child‐Pugh score according to liver fibrosis in hepatocellular carcinoma patients with Child‐Pugh A following hepatectomy

**DOI:** 10.1002/ags3.12498

**Published:** 2021-09-19

**Authors:** Tatsunori Miyata, Yo‐ichi Yamashita, Kota Arima, Takaaki Higashi, Hiromitsu Hayashi, Katsunori Imai, Hidetoshi Nitta, Akira Chikamoto, Toru Beppu, Hideo Baba

**Affiliations:** ^1^ Department of Gastroenterological Surgery Graduate School of Life Sciences Kumamoto University Kumamoto Japan; ^2^ Department of Surgery Saiseikai Kumamoto Hospital Kumamoto Japan; ^3^ Department of Surgery Yamaga City Medical Center Kumamoto Japan

**Keywords:** advanced fibrosis, Child‐Pugh score, HCC, prognosis, The ALBI grade

## Abstract

**Background:**

The albumin‐bilirubin (ALBI) grade was developed to predict the prognosis of patients with hepatocellular carcinoma (HCC), which can stratify the prognosis even in HCC patients with Child‐Pugh A. We evaluated the prognostic efficacy of the ALBI grade and Child‐Pugh classification in HCC patients with Child‐Pugh A stratified by the presence or absence of advanced fibrosis or a preoperative biomarker for advanced fibrosis.

**Methods:**

We retrospectively analyzed 490 consecutive HCC patients with Child‐Pugh A who underwent initial hepatectomies. The accuracy of prognostic prediction using both models was compared by the presence or absence of advanced fibrosis (F3‐4) and its predictor, the preoperative platelet count (PLT).

**Results:**

The prognostic accuracy of the ALBI grade was better in patients without advanced fibrosis (F3‐4; likelihood ratio: 4.39, corrected Akaike information criterion [AICc]: 453.0, *P* = .074), but Child‐Pugh score was better in the advanced fibrosis group (likelihood ratio: 10.67, AICc: 915.2, *P* = .0014). In the high PLT group (≥140 × 10^3^/μL), the prognostic accuracy using the ALBI grade was better in overall survival (OS) and relapse‐free survival (RFS), but in the low PLT group, the Child‐Pugh score was the more accurate model in OS and RFS.

**Conclusions:**

Depending on the degree of fibrosis or preoperative PLT, the ALBI grade and Child‐Pugh score may provide more accurate prognoses after initial hepatectomy in HCC patients with Child‐Pugh A.

## Graphical abstract

Depending on the degree of fibrosis and preoperative platelet count (PLT), the albumin‐bilirubin (ALBI) grade or Child‐Pugh score may provide more accurate prognoses after initial hepatectomy in HCC patients with Child‐Pugh A.

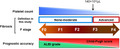

## 1 INTRODUCTION

Hepatocellular carcinoma (HCC), which comprises approximately 90% of liver cancers, is the fourth‐leading deadly cancer worldwide.^1^ Hepatectomy is obviously the most effective treatment to achieve a cure in selected HCC cases; however, 70% of cases have recurrence within 5 years after hepatectomy.^2^, ^3^ Many studies have demonstrated that repeat hepatectomy for recurrent HCC is a promising therapeutic strategy to improve a patient’s prognosis.^4^ For initial and repeat hepatectomy, preoperative liver function is an essential factor to determine an indication of hepatectomy or resectable liver volume for HCC, to allow safe hepatectomy without postoperative complications, including death.^2^, ^5^

The Child‐Pugh classification is one of the most common assessment models for liver function in decision‐making processes for the treatment of HCC worldwide. It was originally developed to evaluate prognoses in patients with portal hypertension and cirrhosis following treatment for variceal bleeding.^6^, ^7^ Because of this background, most patients who were considered for hepatectomy were categorized by Child‐Pugh A; however, it includes a wide range of liver function so that the Child‐Pugh classification does not always correctly reflect the prognosis after hepatectomy.^8^, ^9^, ^10^ Because of these issues, the albumin‐bilirubin (ALBI) grade was developed in 2015, and its prognostic value was validated using several large international cohorts with HCC.^8^, ^11^, ^12^, ^13^ Even in HCC patients with Child‐Pugh A following hepatectomy, the ALBI grade stratified patient prognoses.^8^, ^13^, ^14^ Therefore, the ALBI grade might be a better assessment model for predicting a prognosis than the Child‐Pugh classification.^12^

However, given the history of the development of the Child‐Pugh classification,^6^, ^7^ it might be appropriate for assessing cirrhosis or advanced fibrosis, but not for assessing the prognosis after hepatectomy in HCC patients with relatively mild fibrosis.

We hypothesized that the prognostic accuracy of the ALBI grade and Child‐Pugh score would be different in patients with or without advanced fibrosis. In addition, we thought that it would be useful in clinical practice to use both models to predict prognosis according to the biomarkers of fibrosis before hepatectomy. The aim of this study was to investigate the prognostic accuracy of the ALBI grade and Child‐Pugh score depending on the presence or absence of advanced fibrosis, or its biomarker in HCC patients with Child‐Pugh A following hepatectomy.

## 2 PATIENTS AND METHODS

### 2.1 Patients

Between January 2000 and December 2015, 531 consecutive patients underwent initial and curative hepatectomy for HCC at the Department of Gastroenterological Surgery, Kumamoto University, Kumamoto, Japan. Of these patients, we excluded patients with Child‐Pugh B (n = 18) and those with unavailable pathological fibrosis data (n = 23). Finally, 490 patients were enrolled in this study. The pathologists histologically confirmed all tumors as HCC. Written informed consent was obtained from all patients before treatment. This study was approved by the Human Ethics Review Committee of the Graduate School of Medicine, Kumamoto University, Kumamoto, Japan.

### 2.2 Surgical indications and procedures

Each hepatectomy was performed based on the tumor location, extent of the tumor invasion, parenchymal liver function, and the patient’s general condition, as described previously.^15^ Before hepatectomy, patients underwent liver function tests, including bilirubin, albumin, prothrombin time, and indocyanine green retention rate at 15 minutes. Major hepatectomy was defined as resection of three or more liver segments.

### 2.3 Data collection and patient follow‐up

Preoperative data were collected within 1 month before each hepatectomy. The ALBI grade formula is as follows: ALBI grade = 0.669 log_10_ (total bilirubin [µmol/L]) − 0.085 (albumin [g/L]). The ALBI grade is stratified as grade 1 (−2.60 or less), grade 2 (−2.59 to −1.39), and grade 3 (greater than −1.39).^8^ Tumor stage was assessed according to the 8th edition of the AJCC staging system.^16^ All patients were followed up with a physical examination, determination of α‐fetoprotein (AFP) and des‐γ‐carboxyprothrombin (DCP) levels, and an imaging examination every 3 months for 2 years after hepatectomies, and every half a year thereafter. Recurrence was defined as the appearance of a lesion with radiological features typical of HCC. If recurrence was detected, the patient underwent additional treatments with hepatectomy, local ablation therapy, or transarterial chemoembolization according to recurrence patterns, their general condition, and remnant liver functions. Overall survival (OS) was defined from hepatectomy to death or last follow‐up. Relapse‐free survival (RFS) was defined from hepatectomy to recurrence, death, or last follow‐up. The patients were followed until death or December 31, 2019. Postoperative complications were classified by the Clavien‐Dindo Classification (CD)^17^; we defined CD IIIa or more as a postoperative complication.

### 2.4 Histological assessments

We utilized the New Inuyama Classification^18^ to evaluate histological liver fibrosis. The fibrosis severity (F score) was classified into five subgroups: F0 (no fibrosis); F1 (mild fibrosis), fibrous portal expansion; F2 (moderate fibrosis), bridging fibrosis; F3 (severe fibrosis), bridging fibrosis with distorted acinar architecture; and F4, cirrhosis. All resected specimens were evaluated by pathologists who were blinded to the outcome of patients with HCC. We defined F3 and F4 as advanced fibrosis. If each case was diagnosed as intermediate between each category, it was classified as a severe category (e.g. F4 for F3‐4).

### 2.5 Statistical analysis

Continuous variables were presented as medians (interquartile ranges [IQRs]). Survival outcome was estimated using the Kaplan‐Meier method and compared using the generalized Wilcoxon test. To assess interactions among the preoperative factors, the ALBI grade and Child‐Pugh score were cross‐correlated with another variable of interest via an univariable Cox proportional hazard model, and the interaction was evaluated using the Wald test. We compared the prognostic accuracy of the ALBI grade and Child‐Pugh score by assessing their homogeneity (likelihood ratio chi square values, related to the generalized Wilcoxon test) and discriminatory ability (corrected Akaike information criterion [AICc], related to univariate Cox analyses). More accurate models showed higher likelihood ratio chi square values and lower AICc values. The platelet count threshold was calculated using a receiver operating characteristic (ROC) curve for advanced fibrosis (F3‐4). All results with two‐tailed values of *P* < .05 were considered statistically significant. All statistical analyses were performed using JMP software (Version 12; SAS Institute).

## 3 RESULTS

### 3.1 Patient characteristics

Among 490 HCC patients with Child‐Pugh A, the median age was 67 years, and 382 were men (77.8%). Eighty‐two patients had Child‐Pugh scores of 6 (16.7%), and 195 patients were ALBI grade 2 (39.9%). There were no patients with ALBI grade 3 in this cohort. The numbers of patients classified by the ALBI grade and Child‐Pugh score are shown in Table S1. Histological assessment showed 277 patients (56.5%) had advanced fibrosis (F3‐4). The other perioperative factors and tumor‐related factors are shown in Table 1. In particular, in the association between tumor‐related factors, which have been reported to be associated with prognosis, and the ALBI grade and Child‐Pugh score, AFP was higher in patients with ALBI grade 2 than those with grade 1 (*P* = .0029). On the other hand, there was no significant difference in Child‐Pugh score (Table S2). The median observation period in this cohort was 8.8 years. During the follow‐up of 490 patients, there were 269 recurrences (54.9%) and 144 deaths (29.4%), and the 5‐year OS rate was 73.5%.

**TABLE 1:** The characteristics of this cohort

Variables	All (n = 490)
Clinical factors	
Age (years)	67 (61‐74)
Male	382 (77.8%)
HBs‐Ag positive	128 (26.1%)
HCV‐Ab positive	236 (48.2%)
Total bilirubin (mg/dL)	0.8 (0.6‐1.0)
Albumin (g/dL)	4.0 (3.7‐4.3)
Prothrombin time (%)	95 (86‐104)
Platelet count (×10^3^/µL)^a^	146 (112‐190)
Child‐Pugh score 6	82 (16.7%)
ALBI grade 2	195 (39.9%)
ICG R15 (%)	11.7 (7.8‐16.8)
AFP (ng/mL)	12.8 (4.8‐149)
DCP (mU/mL)	86 (24‐785)
Perioperative factors	
Major hepatectomy	135 (27.6%)
Anatomical resection	351 (71.6%)
Blood loss (ml)	395 (180‐726)
Blood transfusion	64 (13.1%)
Operating time (min)	396 (324‐476)
Postoperative complication (CD ≥ III)	90 (18.4%)
Tumor‐related factors	
Tumor size > 5 (34)	127 (25.9%)
Multiple tumors	139 (28.4%)
Microvascular invasion	181 (36.9%)
Poorly differentiation^b^	100 (20.7%)
Advanced fibrosis (F3‐4)	277 (56.5%)
AJCC Stage I/II/III	239/194/57

Abbreviations: AFP, alpha‐fetoprotein; ALBI, albumin‐bilirubin; CD, Clavian‐Dindo classification; DCP, des‐gamma‐carboxy prothrombin; HBs‐Ag, hepatitis B surface antigen; HCV‐Ab, hepatitis C virus antibody; ICG R15, indocyanine green retention rate at 15 min.

^a^Five patients' data were unavailable. ^b^Seven patients' data were unavailable.

### 3.2 The prognosis according to the ALBI grade and Child‐Pugh score

As many studies have already demonstrated,^8^ in this study, the ALBI grade stratified the prognosis in HCC patients with Child‐Pugh A in OS (*P* = .0003; Figure S1A). The Child‐Pugh score (5 or 6) also stratified the prognosis in OS (*P* <.0001; Figure S1B). In addition, the subgroup analysis showed that the ALBI grade and Child‐Pugh score were significant prognostic factors in OS, but each preoperative factor did not modify the risk in OS in both models (Figure S2). For RFS, as well as OS, the ALBI grade (*P* = .0018) and the Child‐Pugh score (*P* = .0039) also stratified the prognosis in HCC patients with Child‐Pugh A (Figure S1C,D).

### 3.3 The ALBI grade in patients with Child‐Pugh score 5

Next, we examined whether the ALBI grade could stratify patients with the most favorable liver function, classified as Child‐Pugh score 5 (n = 408). Patient characteristics related to liver function (total bilirubin, albumin, prothrombin) were significantly worse in the ALBI grade 2 group (n = 122) than in the ALBI grade 1 group (n = 286). In addition, in the ALBI grade 2 group, the platelet count was lower (135 × 10^3^/µL vs 157 × 10^3^/µL, *P* = .021), the AFP level was higher (17.2 ng/mL vs 10.2 ng/mL, *P* = .018), the rates of anatomical resection were lower (64.8% vs 76.6%, *P* = .018), and advanced fibrosis was higher (63.1% vs 49.0%, *P* = .018) than those in the ALBI grade 1 group (Table 2). However, there was no significant difference in OS (*P* = .074) and RFS (*P* = .076) between the two groups (Figure S3A,B). Median survival time in the grade 1 and grade 2 groups was 11.0 and 8.4 years in OS, 3.6 and 1.9 years in RFS, respectively.

**TABLE 2:** The clinicopathological characteristics, tumor‐related factors, and perioperative factors of patients with Child‐Pugh score 5 (n = 408) according to the ALBI grade

Variables	ALBI		*P*
	Grade 1 (n = 286)	Grade 2 (n = 122)	
Clinical factors			
Age (y)	7.5 (61‐74)	67.0 (59‐74)	.52
Male	228 (79.7%)	88 (72.1%)	.12
HBs‐Ag positive	74 (25.9%)	35 (28.7%)	.63
HCV‐Ab positive	125 (43.7%)	61 (50.0%)	.28
Total bilirubin (mg/dL)	0.7 (0.6‐0.9)	0.9 (0.7‐1.1)	<.0001
Albumin (g/dL)	4.3 (4.1‐4.5)	3.7 (3.7‐3.9)	<.0001
Prothrombin time (%)	99 (91‐106)	83 (91‐98)	<.0001
Platelet count (×10^3^/µL)^a^	157 (123‐193)	135 (107‐179)	.**021**
ICG R15 (%)	10.3 (6.8‐13.7)	14.7 (9.2‐21.0)	<.0001
AFP (ng/mL)	10.2 (4.0‐144)	17.2 (7.0‐149)	.**018**
DCP (mU/mL)	75 (23‐584)	109 (24‐1169)	.52
Perioperative factors			
Major hepatectomy	83 (29.0%)	35 (28.7%)	1.0
Anatomical resection	219 (76.6%)	79 (64.8%)	.**015**
Blood loss (mL)	380 (173‐736)	381 (180‐674)	.79
Blood transfusion	26 (9.1%)	16 (13.1%)	.29
Operating time (min)	399 (327‐467)	397 (311‐466)	.61
Postoperative complication (CD ≥ III)	51 (17.8%)	18 (14.8%)	.48
Tumor‐related factors			
Tumor size > 5 (cm)	78 (27.3%)	27 (22.1%)	.32
Multiple tumors	79 (27.6%)	37 (30.3%)	.63
Microvascular invasion	115 (40.2%)	38 (31.2%)	.094
Poorly differentiation^b^	60 (21.1%)	21 (17.4%)	.42
Advanced fibrosis (F3‐4)	140 (49.0%)	77 (63.1%)	.**0094**
AJCC Stage I/II/III	136/114/36	63/47/12	.64

Abbreviations: AFP, alpha‐fetoprotein; ALBI, albumin‐bilirubin; DCP, des‐gamma‐carboxy prothrombin; HBs‐Ag, hepatitis B surface antigen; HCV‐Ab, hepatitis C virus antibody; ICG R15, indocyanine green retention rate at 15 min.

^a^Five patients' data were unavailable. ^b^Three patients' data were unavailable.

### 3.4 Preoperative prognostic efficacy of the ALBI grade and Child‐Pugh score according to the presence or absence of advanced fibrosis

Next, to evaluate our hypotheses that the ALBI grade was a more suitable model to predict prognosis in patients with a moderate or low degree of fibrosis (F0‐2), we compared the prognostic accuracy of both models according to the presence or absence of advanced fibrosis (F3‐4).

**FIGURE 1:**
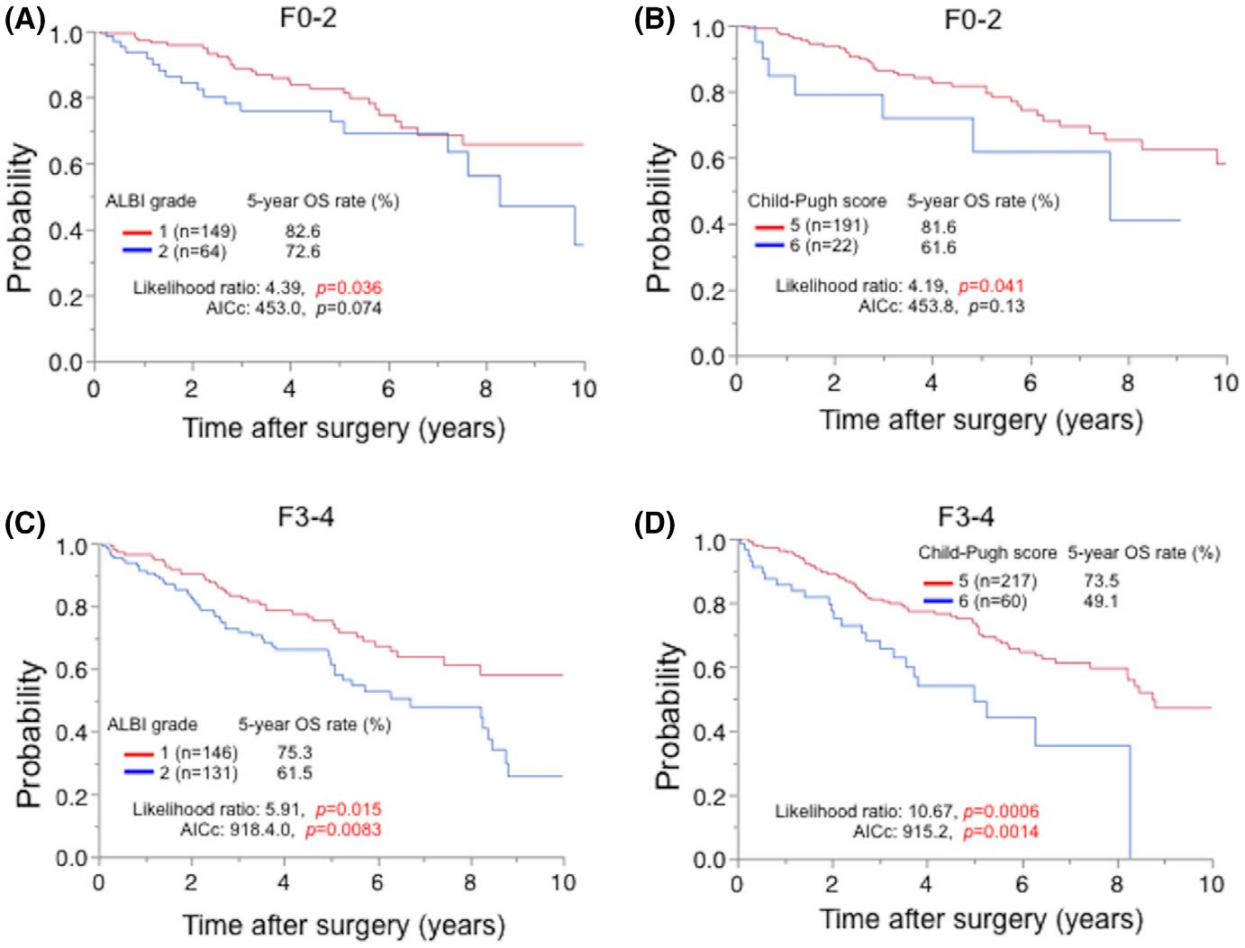
The ALBI grade and the Child‐Pugh score stratified overall survival both in HCC patients with F0‐2 and F3‐4. The prognostic efficacy of the ALBI grade was better in patients with F0‐2 (n = 213) (A, B), whereas that of the Child‐Pugh score was more accurate in those with F3‐4 (n = 277) (C, D). ALBI, albumin‐bilirubin; AICc, Corrected Akaike information criterion; OS, overall survival

In the F0‐2 group (n = 213), the numbers of patients classified into worsened categories by the ALBI grade and Child‐Pugh score were 64 (30.0%) and 22 (10.3%), respectively. The homogeneity (likelihood ratio) and discriminatory ability (AICc) of the ALBI grade were better than those of the Child‐Pugh score (likelihood ratio: 4.39 [*P* = .036] vs 4.19 [*P* = .041], AICc: 453.0 [*P* = .074] vs 453.8 [*P* = .13], ALBI vs Child‐Pugh score; Figure 1A,B).

Next, in the F3‐4 group (n = 277), the numbers of patients classified into worsened categories by each biomarker were 131 (47.3%) by the ALBI grade and 60 (21.7%) by Child‐Pugh score. Contrary to the F0‐2 group, the likelihood ratio and AICc of Child‐Pugh score were better than those of the ALBI grade (likelihood ratio: 5.91 [*P* = .015] vs 10.67 [*P* = .0005], AICc: 918.4 [*P* = .0083] vs 915.2 [*P* = .0014], ALBI vs Child‐Pugh score; Figure 1C,D). Thus, the prognostic accuracy between the ALBI grade and Child‐Pugh score may depend on the presence of advanced fibrosis.

### 3.5 The platelet count level was associated with fibrosis

Next, we evaluated how to apply the characteristics of these two models in clinical practice because they are important to evaluate liver function and prognosis in decision‐making processes for treatment strategies. Accumulating evidence has demonstrated that liver fibrosis is associated with the platelet count.^19^, ^20^ Therefore, we decided to use the preoperative platelet count (PLT) as a biomarker for advanced fibrosis. In the context of the threshold, ROC curve analysis showed 140 × 10^3^/µL was the best to predict advanced fibrosis (AUC: 0.744, *P* < .0001, sensitivity: 65.6%, specificity: 88.3%; Figure S4). Using this threshold, patients were categorized into two groups: those with high PLT (n = 263, 54.2%) or low PLT (n = 222, 45.8%).

### 3.6 Preoperative prognostic efficacy of the ALBI grade and Child‐Pugh classification according to the preoperative platelet count level

**FIGURE 2:**
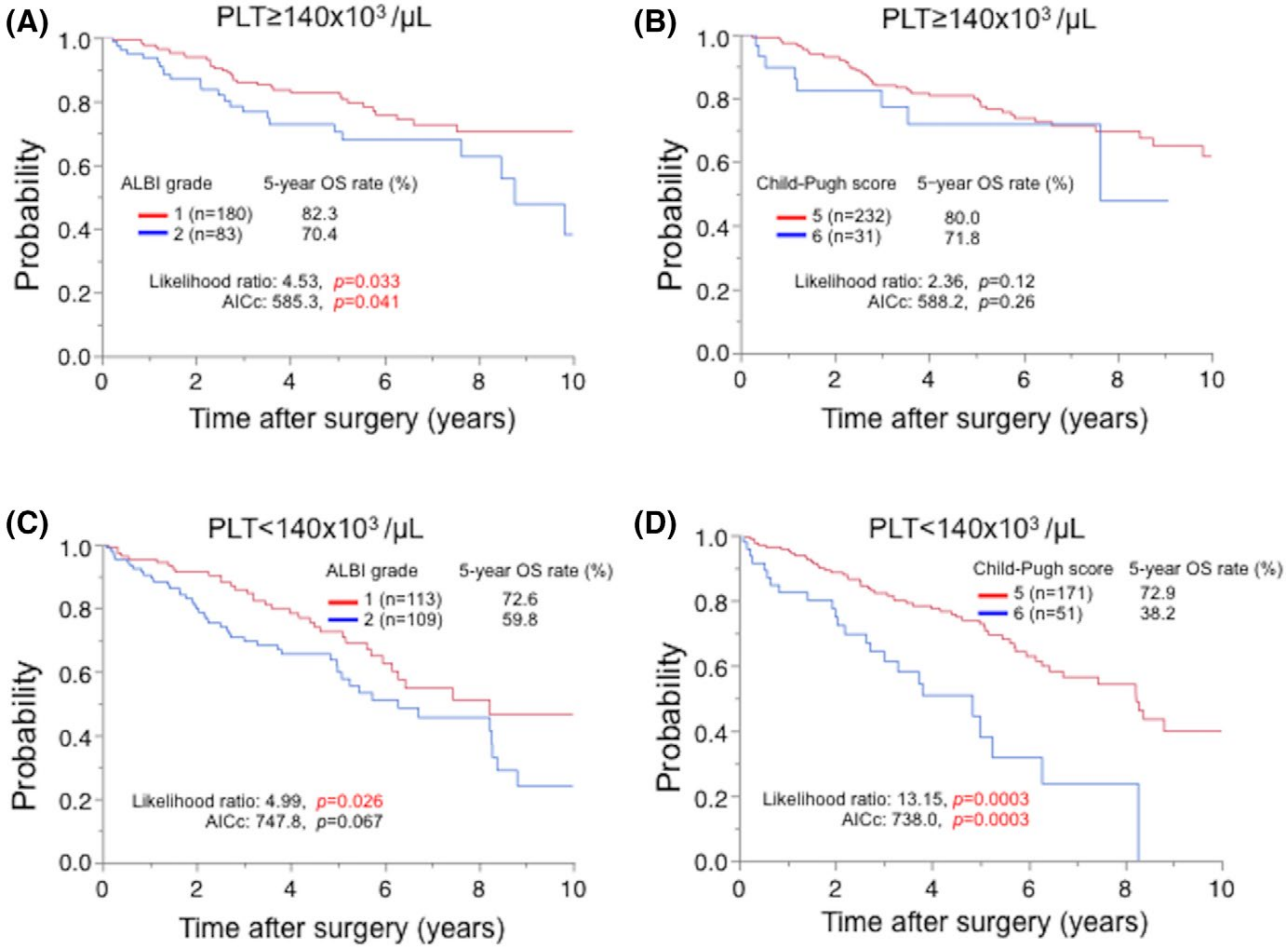
The different prognostic efficacy of the ALBI grade and the Child‐Pugh score was shown according to the platelet count (PLT). The prognostic efficacy of the ALBI grade was better in patients with PLT ≥ 140 x 10^3^/µL (n = 263) (A, B), whereas that of the Child‐Pugh score was better in those with PLT < 140 x 10^3^/µL (n = 222) (C, D). Five patients’ data were unavailable. ALBI, albumin‐bilirubin; AICc, Corrected Akaike information criterion; OS, overall survival

For the OS, in the high PLT group (PLT ≥ 140 × 10^3^/µL), the numbers of patients classified into worsened categories by the ALBI grade and Child‐Pugh score were 83 (31.6%) and 31 (11.8%), respectively. The likelihood ratio and AICc of the ALBI grade were better than those of the Child‐Pugh score (likelihood ratio: 4.53 [*P* = .033] vs 2.36 [*P* = .12], AICc: 585.3 [*P* = .041] vs 588.2 [*P* = .26], ALBI vs Child‐Pugh score; Figure 2A,B). In the low PLT group (PLT < 140 × 10^3^/µL), the numbers of patients classified into worsened categories by the ALBI grade and Child‐Pugh score were 109 (49.1%) and 51 (23.0%), respectively. The likelihood ratio and AICc of the Child‐Pugh score were better than those of the ALBI grade (likelihood ratio: 4.99 [*P* = .026] vs 13.15 [*P* = .0003], AICc: 747.8 [*P* = .067] vs 738.0 [*P* = .0003], ALBI vs Child‐Pugh score; Figure 2C,D).

For RFS, as with the OS, the likelihood ratio for the ALBI grade and AICc were better than those for Child‐Pugh score in the high PLT group (likelihood ratio: 10.3 [*P* = .013] vs 5.05 [*P* = .025]; AICc: 1386.0 [*P* = .0003] vs 1395.5 [*P* = .065], ALBI versus Child‐Pugh score; Figure S5A,B). In the low PLT group, the likelihood ratio and AICc of the Child‐Pugh score were better than those of the ALBI grade, although not significantly different in each model (likelihood ratio: 0.45 [*P* = .50] vs 1.96 [*P* = .16]; AICc: 1331.8 [*P* = .55] vs 1329.4 [*P* = .093], ALBI vs Child‐Pugh score; Figure S5C,D). Thus, the ALBI grade and Child‐Pugh score may have different prognostic accuracy depending on the preoperative PLT.

## 4 DISCUSSION

To the best of our knowledge, this is the first study to assess the prognostic accuracy of the ALBI grade and Child‐Pugh score in HCC patients with Child‐Pugh A following hepatectomy depending on the presence of advanced fibrosis. Our data suggested that the preoperative prognostic efficacy of the ALBI grade and Child‐Pugh score varied depending on the presence of advanced fibrosis (F3‐4). In addition, considering the clinical application of the results of this analysis, we also demonstrated that it might be important to use the models differently depending on the preoperative PLT. Taken together, our results suggested the prognostic value of both models might be affected depending on the status of liver fibrosis.

**FIGURE 3:**
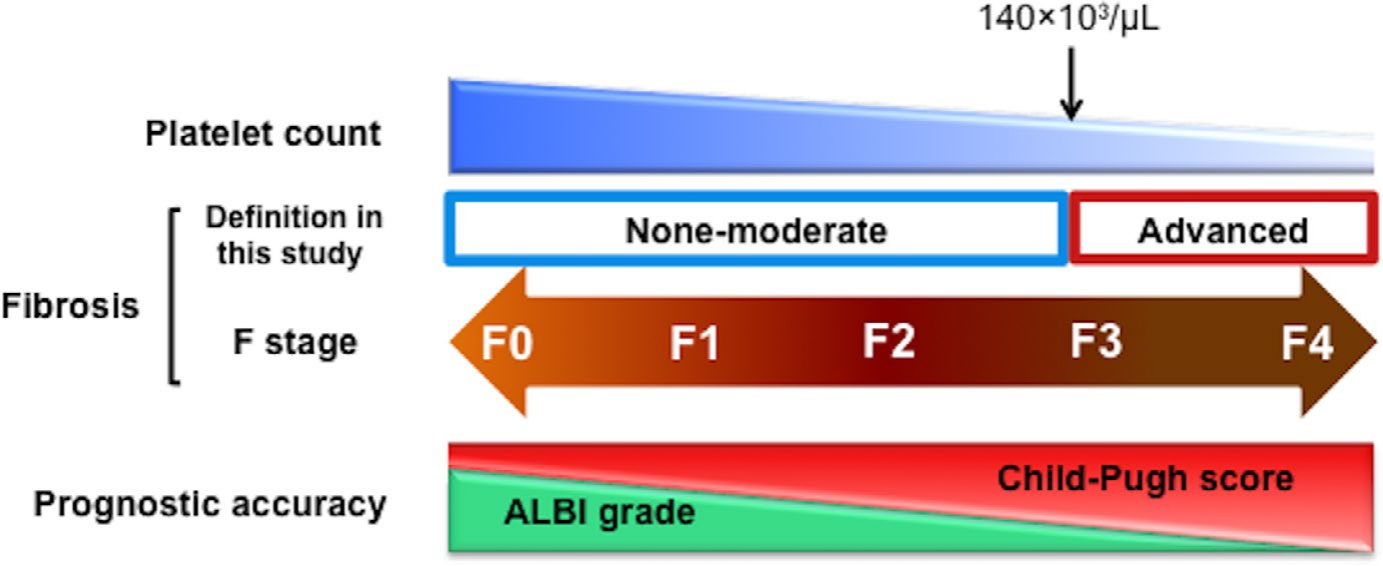
The platelet count, a biomarker of fibrosis, may make a difference in the prognostic accuracy of the ALBI grade and the Child‐Pugh classification. ALBI, albumin‐bilirubin

In this study, we found that the ALBI grade had better prognostic efficacy than the Child‐Pugh score in HCC patients without advanced fibrosis (F0‐2) or with a PLT ≥ 140 × 10^3^/µL (Figures 2 and 3). This indicated that for patients with preserved liver function, the Child‐Pugh score was not able to determine a liver injury, whereas the ALBI grade may be able to identify such liver dysfunction. This may be due to the wide range of changes in each parameter that affects the Child‐Pugh score. In addition, Child‐Pugh includes subjective evaluations. In this study, both OS and RFS could not be stratified by the ALBI grade in patients with Child‐Pugh score 5 (Figure S3); however, in view of the survival curve, it seems necessary to increase the number of patients to verify the results. The ALBI grade might detect patients with relatively low liver function and even those with a Child‐Pugh score of 5 (Table 2), suggesting it may be able to predict patients' prognoses.

Of note, the ALBI grade was not always better than the Child‐Pugh score. In patients with advanced fibrosis or a PLT < 140 × 10^3^/µL, Child‐Pugh score had a better prognostic efficacy after hepatectomy than the ALBI grade. Interestingly, the differences in homogeneity and discriminatory ability between the ALBI grade and Child‐Pugh score in patients with advanced fibrosis or a PLT < 140 × 10^3^/µL were more obvious than in patients without advance fibrosis (F0‐2) or with a PLT ≥ 140 × 10^3^/µL. This suggested that the Child‐Pugh score more accurately sorted patients with a poor prognosis than the ALBI grade in patients with advanced fibrosis. This may be due to the large value range of each parameter leading to an altered Child‐Pugh score, which may increase the sensitivity of the Child‐Pugh score as well as the specificity as fibrosis progresses. Taken together, the ALBI grade may identify prognostic hepatic dysfunction earlier, but as fibrosis progresses, Child‐Pugh score may identify more prognostic hepatic dysfunction in patients with Child‐Pugh A (Figure 3).

Our results also demonstrated that the ALBI grade identified patients with liver disorders that were not detected by the Child‐Pugh score (Table 2). Indeed, only six patients (11.0%) were ALBI grade 1 in patients with a Child‐Pugh score of 6. However, of patients with a Child‐Pugh score of 5, 70.1% were classified as ALBI grade 1 (Table S1), suggesting a Child‐Pugh score of 5 and 6 indicated a different category of liver function when compared with the ALBI grade even though they are in the same category in Child‐Pugh classification. In addition, the ALBI grade could stratify HCC patients with a Child‐Pugh score of 5 in the assessment of liver function and fibrosis (Table 2). Thus, HCC patients with Child‐Pugh A themselves were a heterogeneous group that included a variety of liver functions.

This study also showed that the patients with the ALBI grade 2 displayed higher levels of AFP than those with ALBI grade 1, but this difference was not seen in the Child‐Pugh score (Table S2). Meanwhile, contrary to our result, the two previous studies demonstrated that AFP was not significantly different according to the ALBI grade in patients with Child‐Pugh A.^21^, ^22^ Thus, most tumor‐related factors may be independent of the ALBI grade and Child‐Pugh score; however, to conclude, the association between the ALBI grade and AFP seems to be difficult now because the number of patients was not enough.

Liver biopsy is the gold standard for diagnosing fibrosis, but it is not performed routinely to evaluate fibrosis because of its notable risks.^23^ In this study, we applied PLT as a non‐invasive biomarker to identify advanced fibrosis.^20^, ^24^ Udell et al^20^ demonstrated a PLT of 160 × 10^3^/µL was the most accurate threshold to distinguish cirrhosis with a sensitivity of 0.74 and a specificity of 0.88. Bashour et al^24^ also demonstrated that patients with cirrhosis or advanced fibrosis had a mean platelet level of 144.6 × 10^3^/µL. Compared with previous studies, our cohort included more patients with hepatitis B or C virus (n = 364, 72.3%), which might have had an impact on the lower PLT.^25^ Therefore, our threshold of PLT 140 × 10^3^/µL for advanced fibrosis might be reasonable. Recently, useful serum biomarkers for fibrosis have been identified including the fibrosis‐4 (FIB‐4) index, the aspartate transaminase to platelet ratio index (APRI), and the Mac‐2 binding protein glycosylation isomer.^23^, ^26^, ^27^ Imaging tests can also predict fibrosis.^28^ We were unable to examine patients using these interesting serum or imaging biomarkers; however, the application of them may better reflect our proposed concept of using different biomarkers to assess key liver functions depending on the degree of fibrosis.

This study had several limitations. First, this study was retrospective in design. Second, we did not have enough data on preoperative blood test data such as AST and ALT, preoperative diagnostic imaging for liver fibrosis, the therapies used to treat hepatitis or recurrent HCC, and causes of death. Third, we applied the New Inuyama Classification for the histological evaluation of fibrosis, which was not a common tool worldwide. However, this study was performed in a single institution without bias of surgical procedure with 490 patients with HCC. Therefore, our results might have an important impact on clinical practice.

In conclusion, the ALBI grade and Child‐Pugh score were useful prognosis prediction biomarkers for HCC patients following curative hepatectomy. It may be necessary to use each assessment model while taking into account the level of PLT or degree of fibrosis. Further studies using large‐scale cohorts are needed to validate our findings.

## DISCLOSURE

Funding: This study was supported by the Japan Society for the Promotion of Science (JSPS), 201960331 and 20K16418 (TM).

Conflict of Interest: The authors have no conflicts of interest.

Author Contributions: Conception and design were contributed by TM, KA, TH. Collection and assembly of data were contributed by TM, YY, KA, TH, HH, HN, KI, AC, and TB. Data analysis and interpretation were contributed by TM, AK, TH. Manuscript writing was contributed by TM, YY, and HB. All authors gave final approval of the manuscript.

Ethical Statement: Written informed consent was obtained from all patients before treatment. This study was approved by the Human Ethics Review Committee of the Graduate School of Medicine, Kumamoto University, Kumamoto, Japan.

## Supporting information

Figure S1

Figure S2

Figure S3

Figure S4

Figure S5

Table S1

Table S2
