# Supplementary figures and images for: Alteration of prognostic efficacy of albumin‐bilirubin grade and Child‐Pugh score according to liver fibrosis in hepatocellular carcinoma patients with Child‐Pugh A following hepatectomy
Source: Ann Gastroenterol Surg. 2021 Sep 19;6(1):127–34. doi: 10.1002/ags3.12498 (PMC8786693; doi:10.1002/ags3.12498)

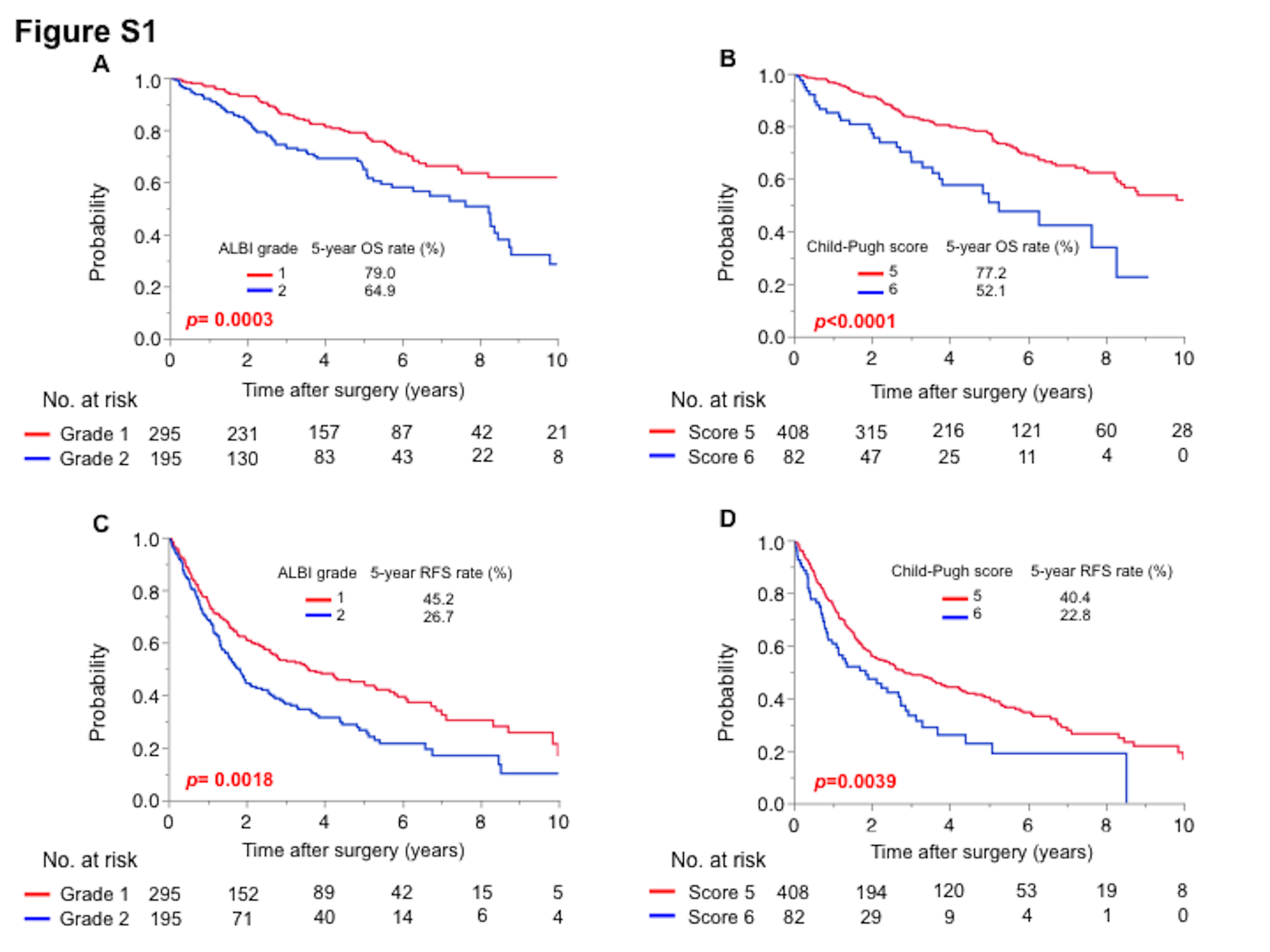

Supplement: Supplementary file 1 — Figure S1 [file AGS3-6-127-s004.tiff]

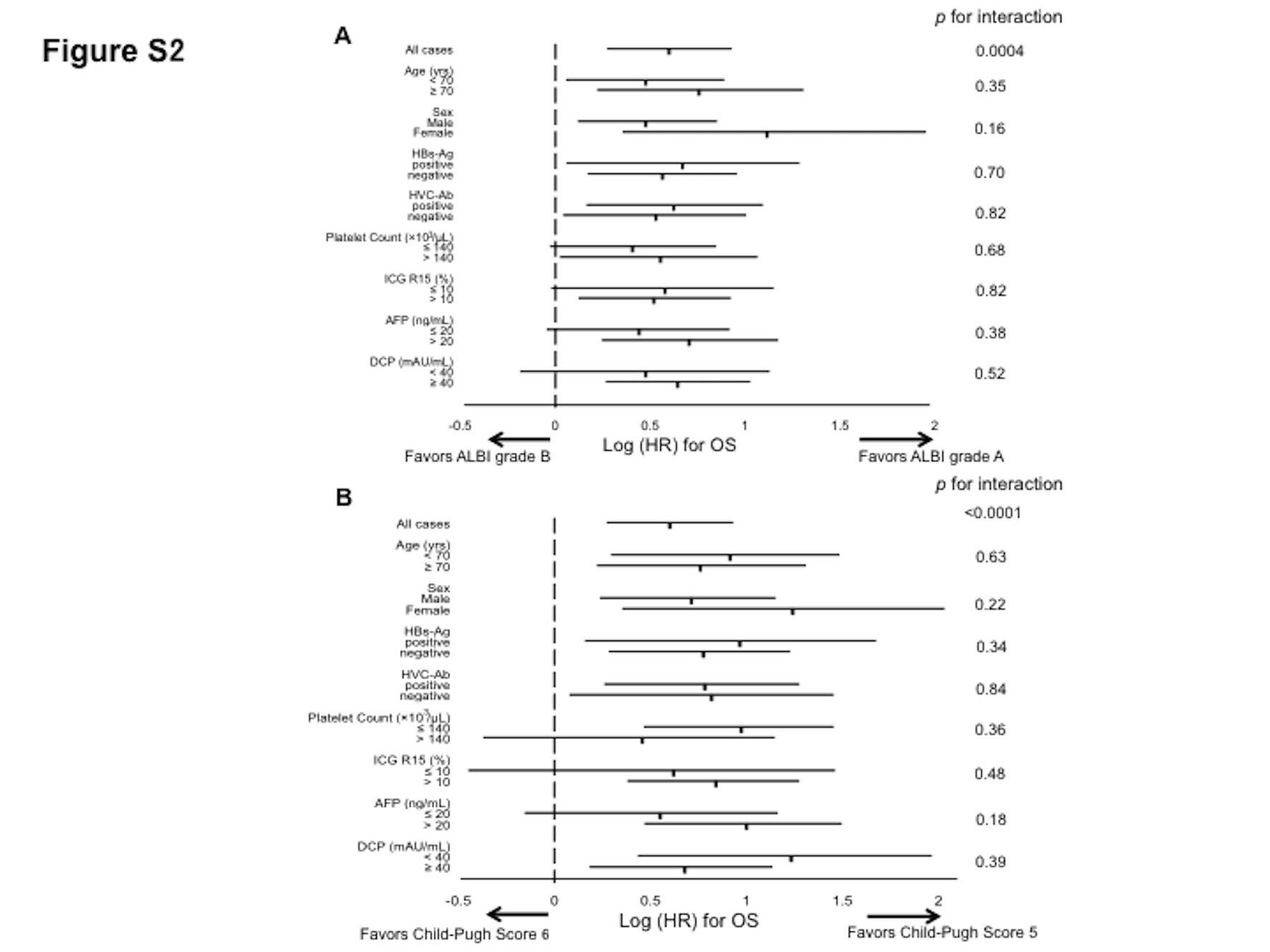

Supplement: Supplementary file 2 — Figure S2 [file AGS3-6-127-s002.tiff]

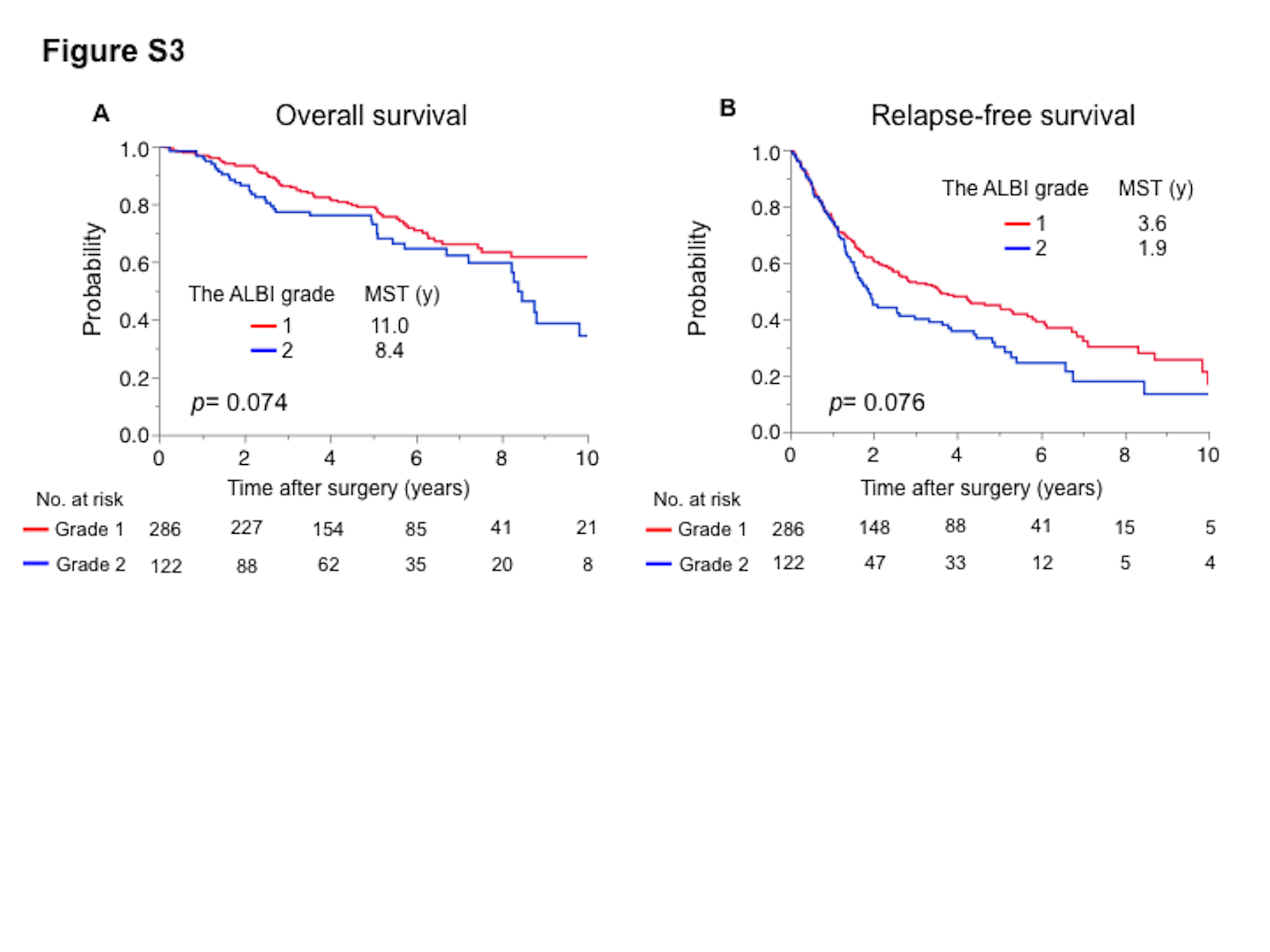

Supplement: Supplementary file 3 — Figure S3 [file AGS3-6-127-s003.tiff]

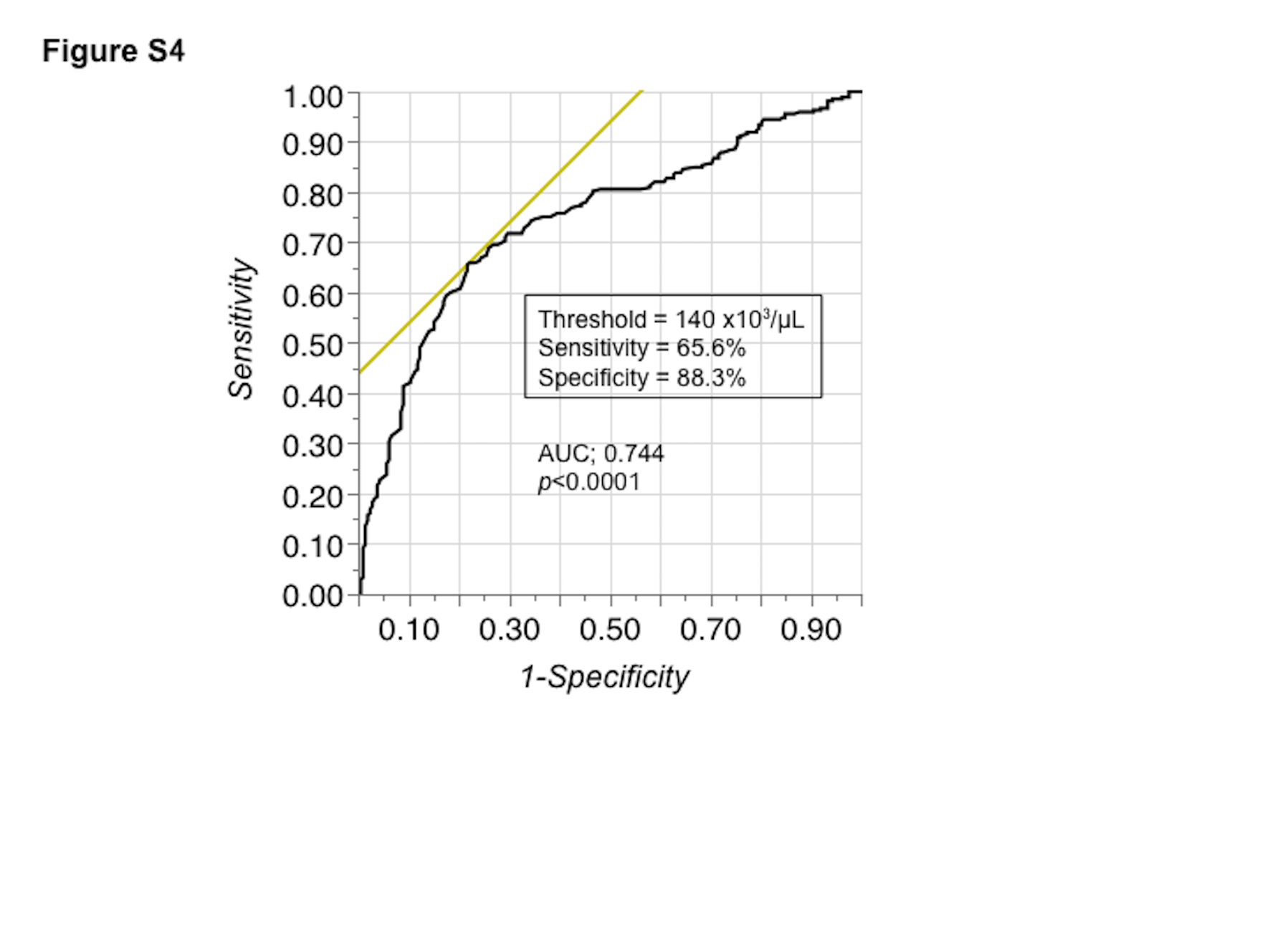

Supplement: Supplementary file 4 — Figure S4 [file AGS3-6-127-s001.tiff]

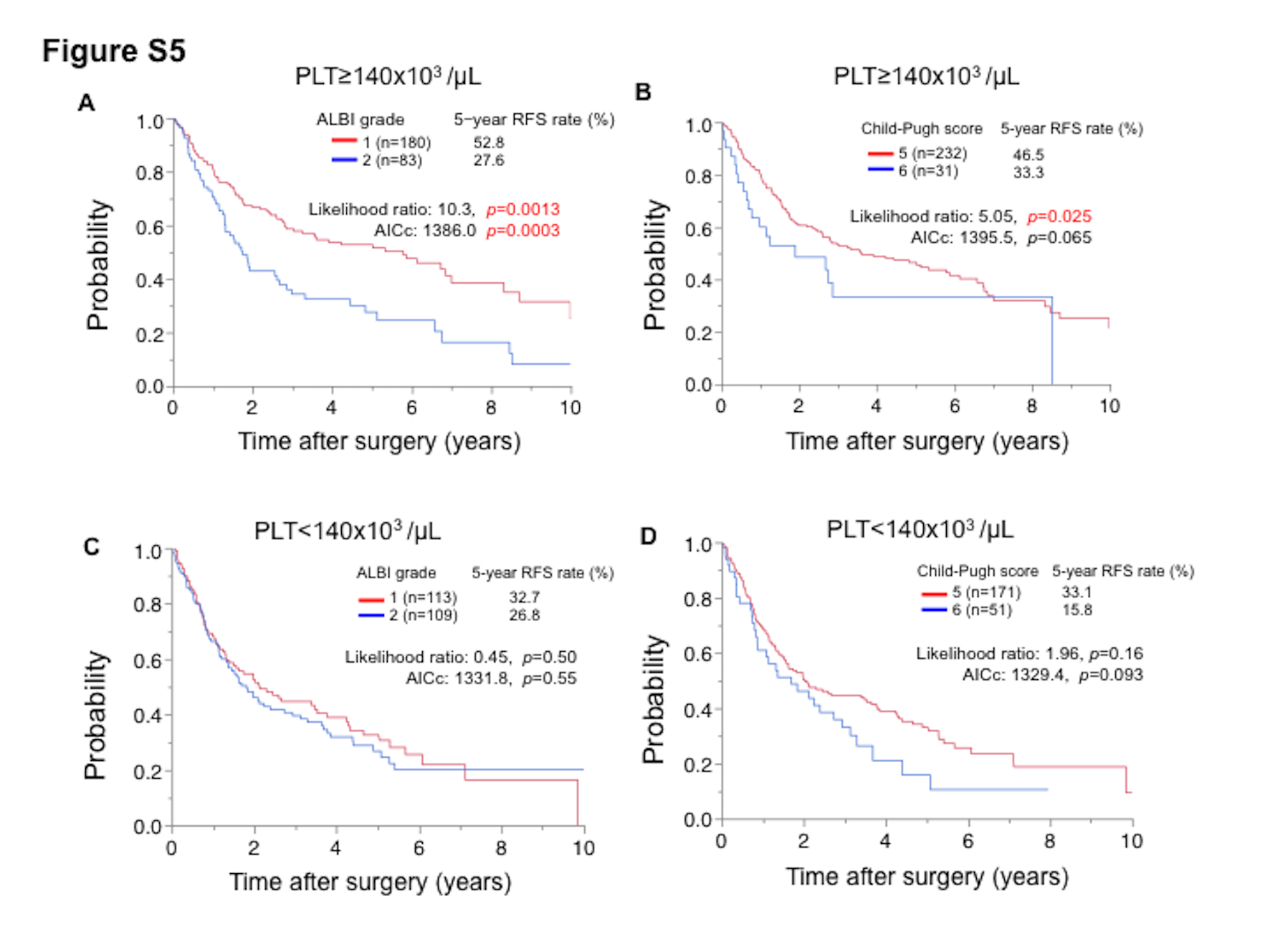

Supplement: Supplementary file 5 — Figure S5 [file AGS3-6-127-s005.tiff]
