# Supplementary material for: Alteration of prognostic efficacy of albumin‐bilirubin grade and Child‐Pugh score according to liver fibrosis in hepatocellular carcinoma patients with Child‐Pugh A following hepatectomy
Source: Ann Gastroenterol Surg. 2021 Sep 19;6(1):127–34. doi: 10.1002/ags3.12498 (PMC8786693; doi:10.1002/ags3.12498)
Supplement: Supplementary file 6 — Table S1 [file AGS3-6-127-s007.docx]

| ***Supplemental Table 1*** The patient number according to the ALBI grade or Child-Pugh score | | |
| --- | --- | --- |
|  | Child-Pugh | |
| ALBI | Score 5 (n=408) | Score 6 (n=82) |
| Grade 1 (n=295) | 286 | 9 |
| Grade 2 (n=195) | 122 | 73 |
| Abbreviations; ALBI, Albumin-bilirubin | | |
