# Supplementary material for: Alteration of prognostic efficacy of albumin‐bilirubin grade and Child‐Pugh score according to liver fibrosis in hepatocellular carcinoma patients with Child‐Pugh A following hepatectomy
Source: Ann Gastroenterol Surg. 2021 Sep 19;6(1):127–34. doi: 10.1002/ags3.12498 (PMC8786693; doi:10.1002/ags3.12498)
Supplement: Supplementary file 7 — Table S2 [file AGS3-6-127-s006.docx]

| ***Supplemental Table 2*** The patients' tumor-related characteristics according to the ALBI grade or Child-Pugh score | | | | | | | |
| --- | --- | --- | --- | --- | --- | --- | --- |
| Variables | ALBI | | *p* |  | Child-Pugh | | *p* |
|  | Grade 1 (n=295) | Grade 2 (n=195) |  |  | Score 5 (n=408) | Score 6 (n=82) |  |
| AFP (ng/mL) | 9.4 (4.0-142) | 18.3 (7.1-154) | 0.0029 |  | 12.3 (4.5-148) | 17.5 (7.1-226) | 0.10 |
| DCP (mU/mL) | 79 (23-599) | 111 (24-1360) | 0.30 |  | 82 (23-683) | 129 (28.8-1747) | 0.20 |
| Tumor size >5 (cm) | 81 (27.5%) | 46 (23.6%) | 0.35 |  | 105 (25.7%) | 22 (26.8%) | 0.89 |
| Multiple tumors | 80 (27.1%) | 59 (30.3%) | 0.47 |  | 116 (28.4%) | 23 (28.0%) | 1.0 |
| Micro vascular invasion | 119 (40.3%) | 62 (31.8%) | 0.057 |  | 153 (37.5%) | 28 (34.2%) | 0.62 |
| Poorly differentiation | 63 (21.5%) | 37 (19.5%) | 0.65 |  | 81 (20.0%) | 19 (24.4%) | 0.45 |
| AJCC Stage I/ II / III | 142/116/37 | 97/78/20 | 0.74 |  | 199/161/48 | 40/33/9 | 0.98 |

Abbreviations; ALBI, Albumin-Bilirubin; AFP, Alpha-fetoprotein; DCP, Des-gamma-carboxyprothrombin
